# Rupture of a subungual glomus tumor of the finger

**DOI:** 10.1186/s12885-018-4377-7

**Published:** 2018-05-02

**Authors:** Hui Lu, Li Feng Chen, Qiang Chen

**Affiliations:** 1 Department of Hand Surgery, The First Affiliated Hospital, College of Medicine, Zhejiang University, #79 Qingchun Road, Hangzhou, Zhejiang Province People’s Republic of China 310003; 2 Department of Hand Surgery, Zhejiang Province People’s Hospital, #158 Shang Tang Road, Hangzhou, Zhejiang province People’s Republic of China 310014; 3 Department of Medical Engineering, The First Affiliated Hospital, Zhejiang University, #79 Qingchun Road, Hangzhou, Zhejiang Province People’s Republic of China 310003

**Keywords:** Subungual glomus tumor, Tumor rupture, Finger infection

## Abstract

**Background:**

Glomus tumor is a rare benign neoplasm, which most frequently occurs in the subungual regions of digits. Tumor rupture and infection occurred in one patient with a glomus tumor have never been reported.

**Case presentation:**

We report a 59-year-old female presented to our hospital with a five-year history of progressively sharp pain and severe tenderness in the tip of her right middle finger. The treatment was surgical excision through a lateral incision accompanied with removal of the nail. After the surgery, the patient gained a functional recovery of her previously afflicted finger.

**Conclusions:**

To the best of our knowledge, this is the first case of finger infection caused by a ruptured subungual glomus tumor. Patients and physicians should be aware of the properties of glomus tumor so that early diagnosis and treatment of subungual glomus tumor as well as avoidance of tumor rupture and infection can be achieved.

## Background

Glomus tumor is a rare benign neoplasm that most frequently occurs in the glomus body in the subungual regions of digits. Glomus tumor represents less than 2% of all soft tissues tumors [1, 2]. The clinical manifestations of glomus tumor were first described as excruciating pain out of proportion to size, localized tenderness, and cold sensitivity by Masson in 1924 [3]. The diagnosis of glomus tumor is made based on both the clinical history and examination. But it is generally difficult to diagnose glomus tumor because of its rarity. After glomus tumor is correctly diagnosed, surgical excision is the most effective treatment [4, 5]. Here, we report a patient with a ruptured and infected subungual glomus tumor in the finger due to a variety of misdiagnoses that delayed the correct diagnosis and effective treatment. To our knowledge, no such case has previously been reported.

## Case presentation

**Fig. 1:**
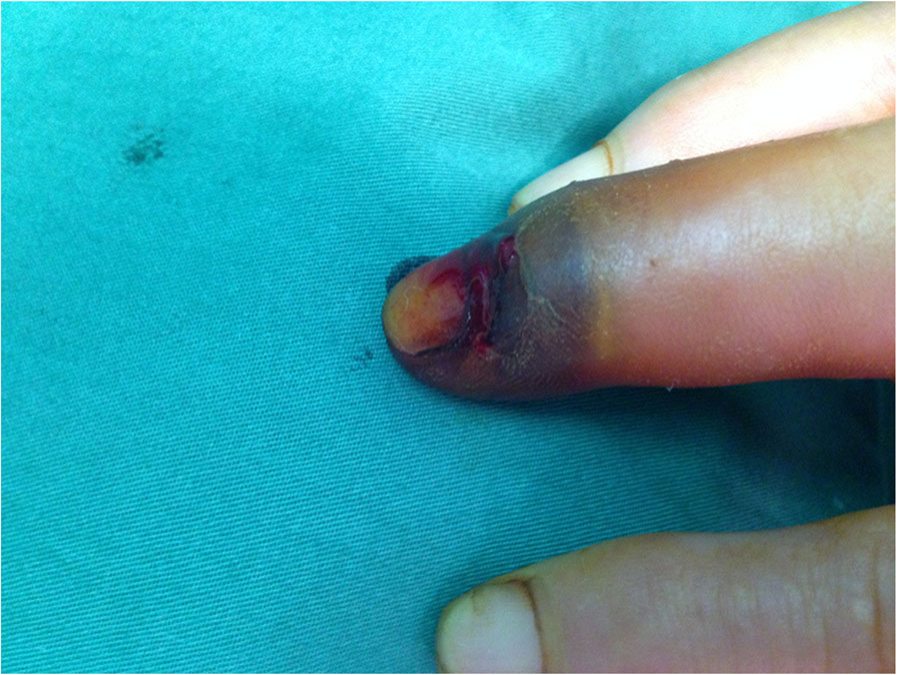
Swelling, pain, and limited motion are observed in the DIP joint. The distal nail root appears dense purple in color

**Fig. 2:**
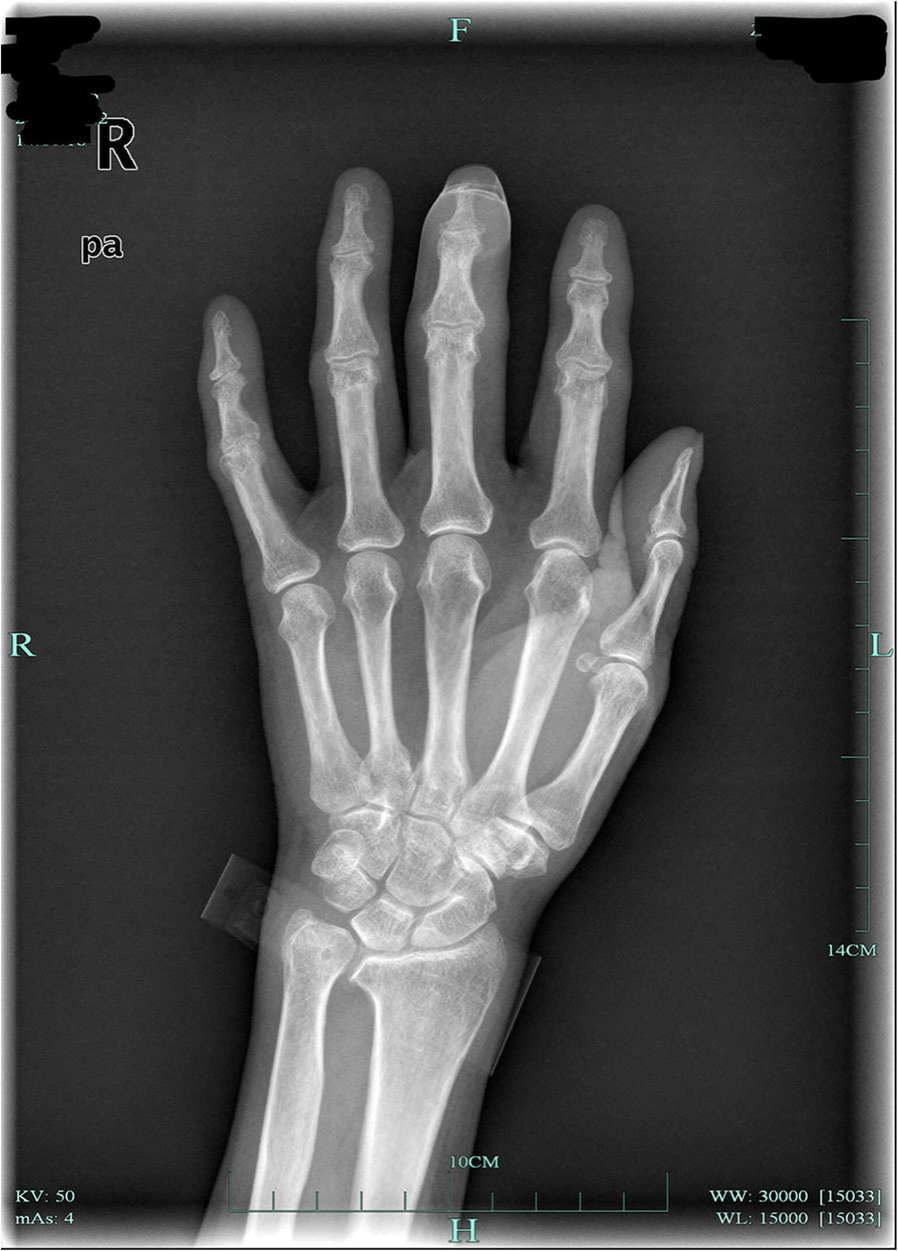
Plain X-ray films document some concavity in the distal phalanx with soft tissue swelling

**Fig. 3:**
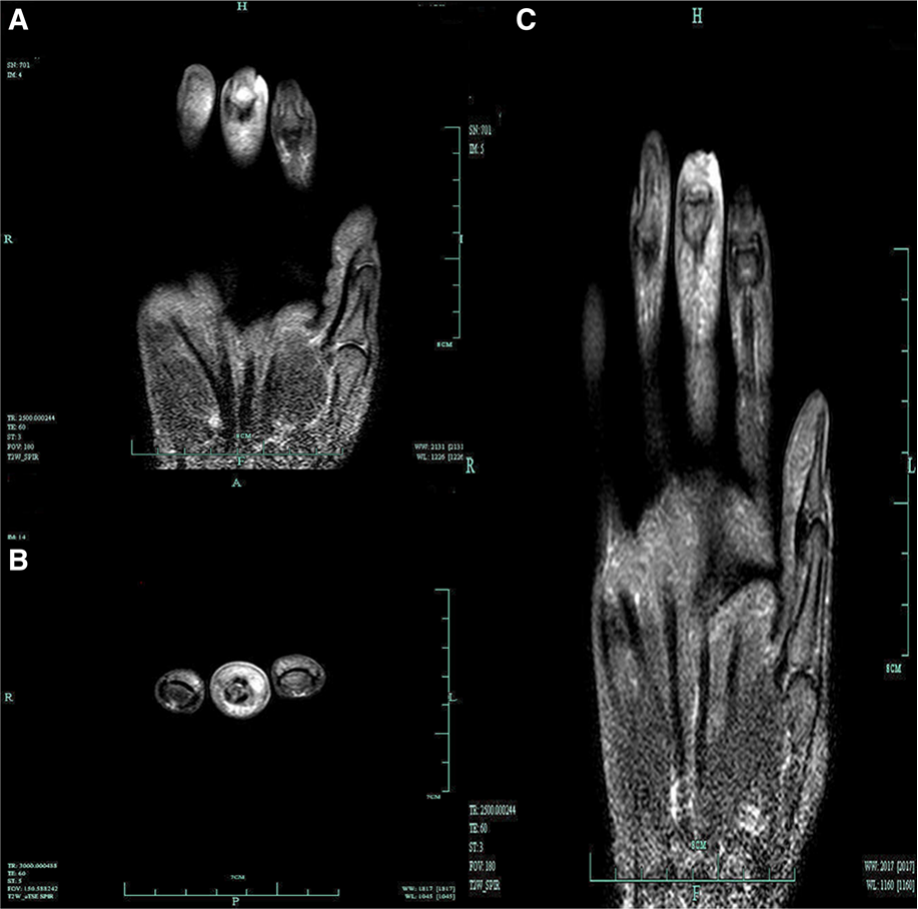
Magnetic resonance imaging (MRI) reveals a subungual approximately solid, well-delimited nodule with hyperintense on the coronal T2-weighted image (**a**) and the axial T2-weighted image (**b**). Soft tissue of the distal middle finger is hyperintense on the coronal T2-weighted image (**c**)

**Fig. 4:**
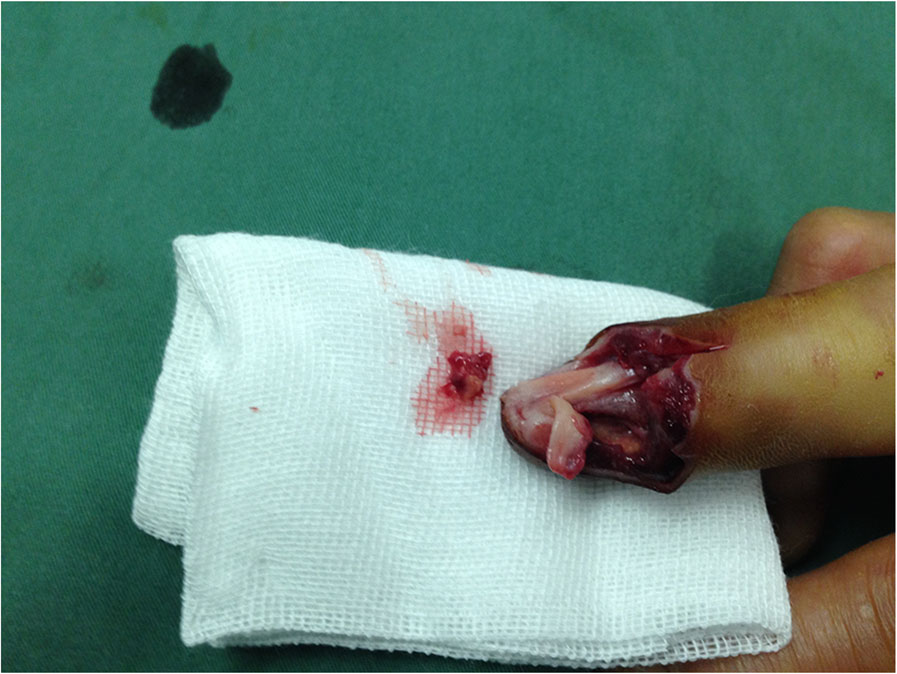
Glomus tumor is ruptured under the nail bed

**Fig. 5:**
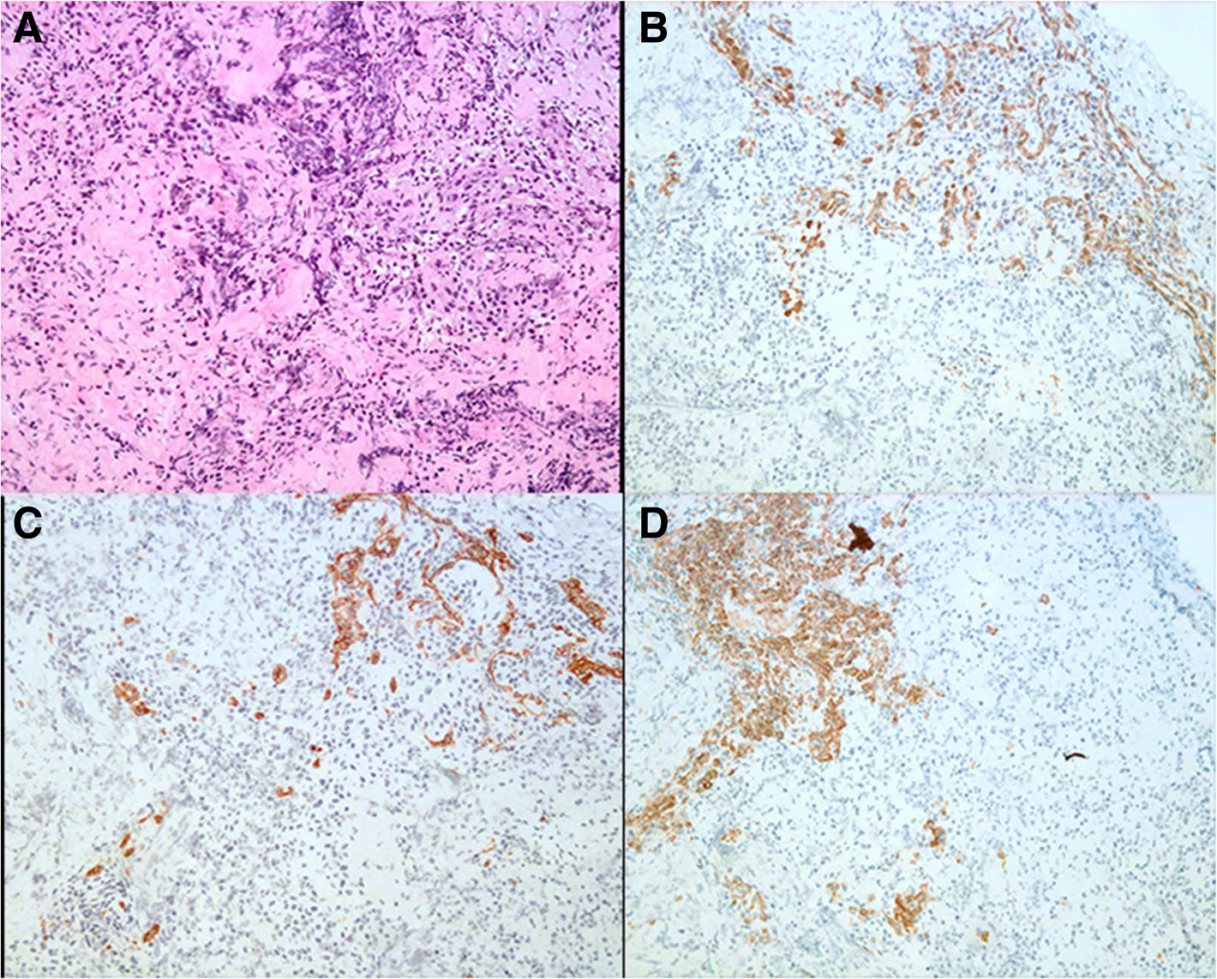
Pathologic examination shows a subungual glomus tumor with chronic inflammatory cell infiltrates (**a**) (200X, HE). Immunohistochemistry shows SMA (+) (**b**), CD34 (+) (**c**), and Caldesmon (+) (**d**)

This is a case of a 59-year-old female presented to our hospital with a five-year history of progressively sharp pain and severe tenderness in the tip of her right middle finger. She had numerous visits to different hospitals in the past, with multiple different diagnoses including neuroma, neuritis, Raynaud’s disease, and menopausal syndrome. She was treated with nonsteroidal anti-inflammatory drugs (NSAID), morphine, and anxiolytics, none of which achieved a response. She even was recommended to receive an amputation of the affected digit. The pain was worse at night or during the heavy physical activity. She was unable to sleep at night due to the worsening pain. Sometimes she forced herself to put finger under her body and sleep on the painful finger. She had no trauma history or prior surgery. Swelling and pain with limited motion were observed in the distal interphalangeal joint (DIP) of the affected finger. The distal nail root appeared dense purple in color due to a long soak in the povidone lodine solution (Fig. 1). Partial necrosis of skin in the nail root was seen. The physical examination revealed the following findings: The distal and middle finger was red, hot, swollen and painful. There were erythema, edema and warmth of the skin in the middle finger. Positive Love’s test, which identified the exact location of the tenderness by pressing with the head of a pin or paper clip. In this particular case, the Love’s test was atypical. The whole finger was tenderness,and the radial subungual of middle finger was more painful. Positive Hildreth’s test, which showed that pain subsided after a tourniquet was applied to the base of the finger. Positive cold sensitivity test, which demonstrated an increase of the localized pain when her middle finger was exposed to cold water, based mainly on the past medical history. Laboratory studies showed that the neutral granular cell count was elevated, but erythrocyte sedimentation rate (ESR) and high-sensitivity C-reactive protein (CRP) were all within the normal ranges. Plain X-ray films documented some concavity in the distal phalanx with soft tissue swelling (Fig. 2). Magnetic resonance imaging (MRI) revealed the bone cortex of distal phalanx in right middle finger was coarse, the boundary was unclear, soft tissue nearby was swollen. An approximately solid, well-delimited subungual nodule with hyperintense on T2-weighted image. Soft tissue of the distal middle finger was swollen with hyperintense on T2-weighted image. (Fig. 3). The lateral, radial and dorsal incision was made according to the appearance of MRI and palpation [6]. We removed the finger nail. The cuticle was partial necrosis. The nail bed and matrix were incised longitudinally, the phalangette was exposed. We found the ruptured glomus tumor, seropurulent pus and inflammatory granulation tissue and we did the debridement in the subungual cavity. The impression of distal phalanx was seen. The nail bed and cuticle were sutured, the wound was closed primarily. (Fig. 4). Surgical procedure was carried out with surgical loupes. Patient who suspected glomus tumor, the clinical diagnosis can be confirmed by MRI and clinical examining (as Love’s test, tourniquet test etc). In this atypical case, we hope to order a tissue biopsy to make a definitive diagnosis. We gave up the biopsy in consideration of pin-track infections. Pathologic findings showed a subungual glomus tumor with glomus cells and chronic inflammatory cell infiltrates. Immunohistochemistry results were SMA (+),desmin (−),CD34 (+),caldesmon (+),vimentin (+) (Fig. 5). Glomus cells are normally immunoreactive with SMA and vimentin. Then the endothelial markers such as CD31 will be helpful in distinguishing glomus tumor from hemangiomas that are lined with unique vascular endothelial cells.

**Fig. 6:**
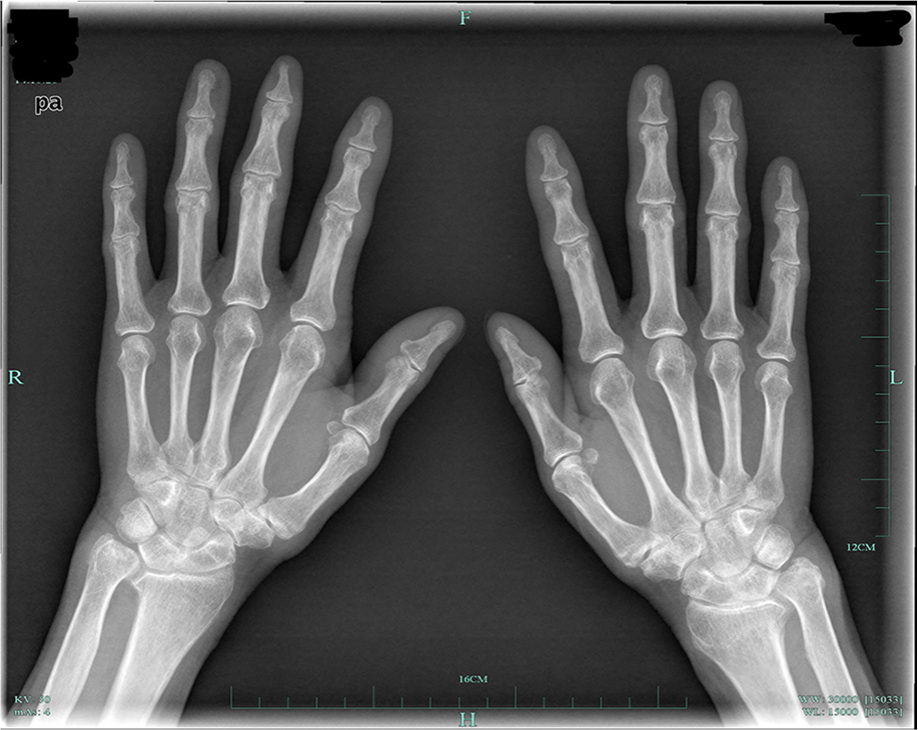
Plain X-ray films document no tumor recurrence in three years after surgery

**Fig. 7:**
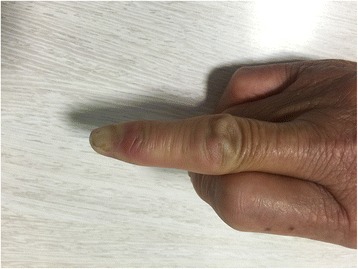
The function and appearance of the affected finger are normal after surgery

After surgery, the patient was pain-free and she reported uninterrupted sleep for the first time in 5 years. She took 200 mg of Celebrex, twice a day for 1 week. The patient received Cefuroxime (Glaxo Wellcome Operations, UK; 0.5 g twice a day) for 1 week after the surgery. She achieved a significant recovery and experienced no tumor recurrence in 3 years after surgery (Figs. 6, and 7). These study protocols were approved by the Medical Ethics Committee of the First Affiliated Hospital, College of Medicine, Zhejiang University.

## Discussion

In authors’ experiences, physical examination and medical history may be informative enough for a correct diagnosis of a disease. However, increased awareness of a disease, especially a rare disease by both the patients and physicians are crucial for early diagnosis and treatment. Delay in the correct diagnosis for this patient presented here eventually led to the rupture and infection of this rare subungual glomus tumor.

Medical imaging can detect the lesion of glomus tumor and thus help arrive at the correct diagnosis. Ultrasound can detect the lesions, but it is so heavily dependent on the operator’s expertise [7]. MRI is a better method for locating and displaying the tumor directly [8, 9], but sometimes it still fails to detect the lesions in some patients in our hospital. In this case, MRI not only detected the tumor, it can also show the infection and the extent of edema in the affected finger.

Surgical resection is the standard treatment for glomus tumor. It is important that surgeons understand the accurate location of the tumor and the regulation of skin circulation to avoid recurrence and nail deformity [10, 11] or necrosis. The dilemma of this case was how to resect the tumor and in the meantime also protect the nail circulation. Based on the MRI image, we chose the lateral incision close to the radial side of the nail to solve the dilemma.

## Conclusion

We have diagnosed the first case of finger infection caused by the rupture of a rare subungual glomus tumor that was initially misdiagnosed multiple times. Increased awareness of this rare tumor by both the patients and physicians may help avoid misdiagnosis and delay of early correct diagnosis and treatment of subungual glomus tumor.

## Abbreviation

MRI: Magnetic resonance imaging
